# 5-Meth­oxy-1-(3,4,5-trimethoxy­phen­yl)-1*H*-indole

**DOI:** 10.1107/S1600536810018568

**Published:** 2010-06-16

**Authors:** Thomas Blake Monroe, Casey Rimland, Yasamin Moazami, Daniel S. Jones, Craig A. Ogle

**Affiliations:** a Department of Chemistry, The University of North Carolina at Charlotte, 9201 University City Blvd, Charlotte, NC 28223, USA

## Abstract

The title compound, C_18_H_19_NO_4_, was prepared as an indole derivative with possible anti­mitotic properties. The planes of the indole and trimethoxy­phenyl rings make a dihedral angle of 45.35 (5)° with one another. In the crystal, mol­ecules related by a twofold screw axis exhibit arene C—H⋯arene-π inter­actions which are 3.035 (1) Å in length.

## Related literature

For a related structure, see: Suthar *et al.* (2005 ▶). For pharmaceutical applications of indoles, see: Fuwa & Sasaki (2009 ▶); Li & Martins (2003 ▶).
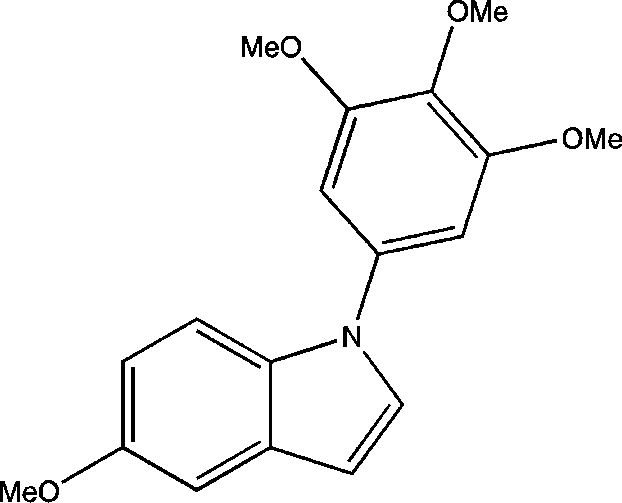


## Experimental

### 

#### Crystal data

C_18_H_19_NO_4_
*M* *_r_* = 313.34
Monoclinic, 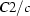

*a* = 19.0036 (16) Å
*b* = 7.3179 (14) Å
*c* = 23.672 (4) Å
β = 96.802 (10)°
*V* = 3268.8 (9) Å^3^
*Z* = 8
Cu *K*α radiation
μ = 0.74 mm^−1^
*T* = 295 K
0.32 × 0.27 × 0.26 mm

#### Data collection

Enraf–Nonius CAD-4 diffractometer
6084 measured reflections
2951 independent reflections
2074 reflections with *I* > 2σ(*I*)
*R*_int_ = 0.026
3 standard reflections every 190 reflections  intensity decay: 4%

#### Refinement

*R*[*F* ^2^ > 2σ(*F* ^2^)] = 0.037
*wR*(*F* ^2^) = 0.110
*S* = 1.00
2951 reflections
209 parameters
H-atom parameters constrained
Δρ_max_ = 0.16 e Å^−3^
Δρ_min_ = −0.16 e Å^−3^

### 

Data collection: *CAD-4 EXPRESS* (Enraf–Nonius, 1994 ▶); cell refinement: *CAD-4 EXPRESS*; data reduction: *XCAD4* (Harms & Wocadlo, 1995 ▶); program(s) used to solve structure: *SHELXS97* (Sheldrick, 2008 ▶); program(s) used to refine structure: *SHELXL97* (Sheldrick, 2008 ▶); molecular graphics: *ORTEP-3 for Windows* (Farrugia, 1997 ▶), *Mercury* (Macrae *et al.*, 2006 ▶); software used to prepare material for publication: *WinGX* (Farrugia, 1999 ▶).

## Supplementary Material

Crystal structure: contains datablocks global, I. DOI: 10.1107/S1600536810018568/fl2291sup1.cif

Structure factors: contains datablocks I. DOI: 10.1107/S1600536810018568/fl2291Isup2.hkl

Additional supplementary materials:  crystallographic information; 3D view; checkCIF report

## Figures and Tables

**Fig. 1.**
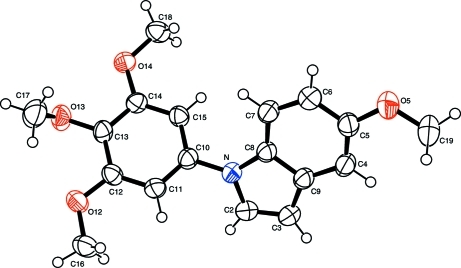
View of the title compound (50% probability displacement ellipsoids)

**Fig. 2.**
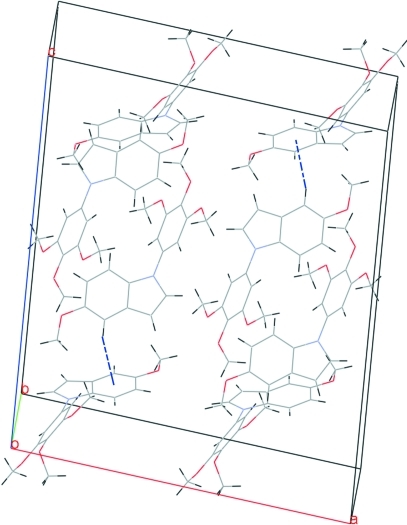
Packing diagram showing the arene C—H···π interactions between molecules related by a two-fold screw axis

**Fig. 3.**
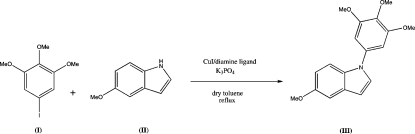
Synthesis scheme

## supplementary crystallographic information

### Comment

The indole core is a common structure observed in a wide variety of biologically active compounds and pharmaceutical products (Li & Martins, 2003). Indole structures are considered as privileged structure motifs, due to their ability to bind many receptors within the body (Fuwa & Sasaki, 2009). As a result, there has been a great deal of research dedicated to incorporating the indole functionality in the design and synthesis of novel anti-mitotic compounds for the treatment of cancer. The title compound was prepared as an indole derivative with possible anti-mitotic properties.

The structure of the title compound is shown in Fig. 1. The plane of the indole ring and the plane of the trimethoxyphenyl ring make a 45.35 (5)° angle with one another. The deviation of methoxy carbon C19 from the indole mean plane is 0.050 (3) Å. The deviations of methoxy carbons C16, C17, and C18 from the plane of the phenyl ring are 0.065 (3) Å, 1.157 (3) Å, and 0.138 (3) Å, respectively. Molecules related by a two-fold screw axis exhibit arene C—H··· arene π interactions, as shown in Fig. 2. The interaction is between C4—H of one molecule and the six membered (C4 through C9) aromatic ring of the screw-related molecule. The H··· ring-centroid distance is 3.035 (1) Å, and the H··· ring-centroid line makes an angle of 5.6 (3)° with the normal to the plane of the ring.

In a comparable structure, 1-(3,4,5-Trimethoxyphenyl)naphthalene (Suthar *et al.*, 2005), the angle between the planes of the napthylene ring and the trimethoxyphenyl ring is 68.19 (10)°.

### Experimental

Preparation of the title compound (III) (See Synthesis scheme): To a Schlenk flask equipped with a magnetic stir bar, 1.47 g (10 mmol) of 5-methoxyindole (II), 6.36 g (30 mmol) of K_3_PO_4_, and 0.190 g (10 mol %) of CuI were added. The reaction flask was then purged with nitrogen gas and charged with 2.94 g (10 mmol) of 5-iodo-1,2,3-trimethoxybenzene (I), 0.22 ml (20 mol %) of *N*,*N*'-dimethylethylenediamine, and 25.0 ml of dry degassed toluene. The reaction mixture was heated to reflux for 24 hours. Upon completion, the crude reaction mixture was filtered through a celite plug, and concentrated on a rotary evaporator to yield an off-white solid. The solid was recrystallized from ethanol to obtain the x-ray quality crystals. Pure product was obtained in 86 % yield (2.70 g). Melting point: 99-101°C. MS(E1): M+ 313 m/z, 298 m/z. 1H NMR (300 MHz, DMSO-d) δ7.62 (d, 1H), 7.56 (d,1*H*), 7.14 (d,1*H*), 6.84(d,1*H*), 6.82 (s, 2H), 6.58 (d, 1H), 3.85 (s,6*H*), 3.78 (s, 3H), 3.71 (s,3*H*)

### Refinement

All H atoms were constrained using a riding model. The aromatic C—H bond lengths were fixed at 0.93 Å, with *U*_iso_(H) = 1.2 *U*_eq_(C). The methyl C—H bond lengths were fixed at 0.96 Å, with *U*_iso_(H) = 1.5 *U*_eq_(C). An idealized tetrahedral geometry was used for the methyl groups, and the torsion angles around the O—C bonds were refined.

### Crystal data

**Table:**

C_18_H_19_NO_4_	*F*(000) = 1328
*M**_r_* = 313.34	*D*_x_ = 1.273 Mg m^−^^3^
Monoclinic, *C*2/*c*	Cu *K*α radiation, λ = 1.54184 Å
Hall symbol: -C 2yc	Cell parameters from 24 reflections
*a* = 19.0036 (16) Å	θ = 6.4–20.8°
*b* = 7.3179 (14) Å	µ = 0.74 mm^−^^1^
*c* = 23.672 (4) Å	*T* = 295 K
β = 96.802 (10)°	Prism, colorless
*V* = 3268.8 (9) Å^3^	0.32 × 0.27 × 0.26 mm
*Z* = 8	

### Data collection

**Table:**

Enraf–Nonius CAD-4 diffractometer	θ_max_ = 67.4°, θ_min_ = 3.8°
non–profiled ω/2θ scans	*h* = −22→22
6084 measured reflections	*k* = −8→0
2951 independent reflections	*l* = −28→28
2074 reflections with *I* > 2σ(*I*)	3 standard reflections every 190 reflections
*R*_int_ = 0.026	intensity decay: 4%

### Refinement

**Table:**

Refinement on *F*^2^	H-atom parameters constrained
Least-squares matrix: full	*w* = 1/[σ^2^(*F*_o_^2^) + (0.0613*P*)^2^ + 0.605*P*] where *P* = (*F*_o_^2^ + 2*F*_c_^2^)/3
*R*[*F*^2^ > 2σ(*F*^2^)] = 0.037	(Δ/σ)_max_ < 0.001
*wR*(*F*^2^) = 0.110	Δρ_max_ = 0.16 e Å^−^^3^
*S* = 1.00	Δρ_min_ = −0.16 e Å^−^^3^
2951 reflections	Extinction correction: *SHELXL97* (Sheldrick, 2008), Fc^*^=kFc[1+0.001xFc^2^λ^3^/sin(2θ)]^-1/4^
209 parameters	Extinction coefficient: 0.00188 (13)
0 restraints	

### Special details

**Table:**

Geometry. All esds (except the esd in the dihedral angle between two l.s. planes) are estimated using the full covariance matrix. The cell esds are taken into account individually in the estimation of esds in distances, angles and torsion angles; correlations between esds in cell parameters are only used when they are defined by crystal symmetry. An approximate (isotropic) treatment of cell esds is used for estimating esds involving l.s. planes.

### Fractional atomic coordinates and isotropic or equivalent isotropic displacement parameters (Å^2^)

**Table:**

	*x*	*y*	*z*	*U*_iso_*/*U*_eq_	
O14	0.15356 (6)	0.12928 (15)	0.49143 (5)	0.0511 (3)	
O13	0.07247 (7)	0.29172 (17)	0.40657 (5)	0.0575 (3)	
O5	0.40130 (7)	0.77324 (19)	0.74372 (5)	0.0647 (4)	
N	0.14855 (7)	0.67340 (19)	0.60827 (5)	0.0451 (3)	
O12	0.02905 (7)	0.64188 (17)	0.41594 (5)	0.0562 (4)	
C11	0.08846 (8)	0.6630 (2)	0.51210 (7)	0.0459 (4)	
H15	0.0741	0.7834	0.5159	0.055*	
C15	0.15105 (8)	0.3970 (2)	0.55189 (6)	0.0429 (4)	
H11	0.1773	0.3391	0.5824	0.051*	
C14	0.13334 (8)	0.3050 (2)	0.50087 (7)	0.0414 (4)	
C8	0.21611 (9)	0.6831 (2)	0.63793 (6)	0.0428 (4)	
C7	0.27934 (9)	0.6005 (2)	0.62755 (7)	0.0488 (4)	
H4	0.2813	0.5235	0.5965	0.059*	
C13	0.09214 (8)	0.3894 (2)	0.45562 (6)	0.0430 (4)	
C12	0.06941 (8)	0.5691 (2)	0.46185 (7)	0.0435 (4)	
C10	0.12910 (8)	0.5758 (2)	0.55666 (6)	0.0427 (4)	
C6	0.33859 (10)	0.6368 (3)	0.66472 (7)	0.0519 (4)	
H3	0.3813	0.5823	0.6589	0.062*	
C5	0.33640 (10)	0.7540 (2)	0.71127 (7)	0.0496 (4)	
C3	0.14176 (10)	0.8679 (2)	0.68041 (7)	0.0539 (5)	
H9	0.124	0.95	0.7052	0.065*	
C18	0.20053 (9)	0.0443 (2)	0.53514 (7)	0.0532 (4)	
H18A	0.2109	−0.0777	0.5237	0.08*	
H18B	0.1786	0.04	0.5696	0.08*	
H18C	0.2437	0.1134	0.5415	0.08*	
C4	0.27495 (10)	0.8393 (2)	0.72137 (7)	0.0520 (4)	
H7	0.274	0.9179	0.7521	0.062*	
C2	0.10450 (10)	0.7872 (2)	0.63464 (7)	0.0512 (4)	
H8	0.0566	0.8057	0.6228	0.061*	
C9	0.21305 (9)	0.8044 (2)	0.68373 (7)	0.0466 (4)	
C16	0.00926 (10)	0.8294 (3)	0.41920 (8)	0.0597 (5)	
H16A	−0.0187	0.8644	0.3844	0.09*	
H16B	0.0511	0.9038	0.4248	0.09*	
H16C	−0.0179	0.8461	0.4505	0.09*	
C17	0.10615 (13)	0.3480 (3)	0.35877 (8)	0.0751 (6)	
H17A	0.0895	0.2739	0.3265	0.113*	
H17B	0.1565	0.3342	0.3673	0.113*	
H17C	0.0951	0.4738	0.3504	0.113*	
C19	0.40555 (13)	0.8959 (3)	0.78968 (9)	0.0857 (7)	
H1A	0.4531	0.8973	0.8086	0.129*	
H1B	0.3736	0.858	0.8159	0.129*	
H1C	0.3929	1.0162	0.7759	0.129*	

### Atomic displacement parameters (Å^2^)

**Table:**

	*U*^11^	*U*^22^	*U*^33^	*U*^12^	*U*^13^	*U*^23^
O14	0.0641 (7)	0.0342 (6)	0.0528 (7)	0.0029 (5)	−0.0026 (6)	−0.0024 (5)
O13	0.0760 (8)	0.0458 (7)	0.0466 (6)	−0.0098 (6)	−0.0091 (6)	−0.0038 (5)
O5	0.0659 (8)	0.0628 (9)	0.0605 (8)	−0.0043 (7)	−0.0125 (6)	−0.0102 (7)
N	0.0516 (8)	0.0402 (8)	0.0423 (7)	0.0020 (6)	0.0006 (6)	−0.0044 (6)
O12	0.0620 (8)	0.0452 (7)	0.0560 (7)	0.0041 (6)	−0.0155 (6)	0.0022 (6)
C11	0.0497 (9)	0.0362 (9)	0.0503 (9)	0.0013 (7)	0.0003 (7)	−0.0013 (7)
C15	0.0465 (9)	0.0387 (9)	0.0420 (8)	−0.0023 (7)	−0.0006 (7)	0.0026 (7)
C14	0.0443 (8)	0.0322 (8)	0.0475 (8)	−0.0049 (7)	0.0050 (7)	0.0006 (7)
C8	0.0522 (9)	0.0359 (9)	0.0394 (8)	−0.0001 (7)	0.0009 (7)	−0.0006 (7)
C7	0.0568 (10)	0.0445 (10)	0.0446 (8)	0.0004 (8)	0.0043 (7)	−0.0065 (7)
C13	0.0468 (9)	0.0386 (9)	0.0421 (8)	−0.0093 (7)	−0.0016 (7)	−0.0018 (7)
C12	0.0409 (8)	0.0398 (9)	0.0480 (9)	−0.0034 (7)	−0.0027 (7)	0.0044 (7)
C10	0.0458 (8)	0.0383 (9)	0.0431 (8)	−0.0040 (7)	0.0021 (7)	−0.0025 (7)
C6	0.0531 (10)	0.0494 (10)	0.0528 (10)	0.0010 (8)	0.0044 (8)	−0.0033 (8)
C5	0.0589 (10)	0.0424 (10)	0.0452 (9)	−0.0040 (8)	−0.0040 (8)	0.0020 (7)
C3	0.0648 (11)	0.0459 (10)	0.0505 (9)	0.0095 (8)	0.0050 (8)	−0.0088 (8)
C18	0.0551 (10)	0.0418 (10)	0.0619 (10)	0.0023 (8)	0.0034 (8)	0.0063 (8)
C4	0.0704 (12)	0.0417 (10)	0.0423 (8)	−0.0013 (9)	−0.0001 (8)	−0.0072 (7)
C2	0.0560 (10)	0.0448 (9)	0.0523 (9)	0.0092 (8)	0.0044 (8)	−0.0017 (8)
C9	0.0608 (10)	0.0371 (9)	0.0409 (8)	0.0014 (8)	0.0022 (7)	−0.0016 (7)
C16	0.0653 (12)	0.0456 (11)	0.0654 (12)	0.0073 (9)	−0.0046 (9)	0.0097 (9)
C17	0.1107 (18)	0.0663 (14)	0.0487 (10)	0.0058 (13)	0.0109 (11)	−0.0032 (10)
C19	0.0995 (17)	0.0828 (17)	0.0663 (13)	0.0018 (14)	−0.0254 (12)	−0.0217 (12)

### Geometric parameters (Å, °)

**Table:**

O14—C14	1.368 (2)	C13—C12	1.397 (2)
O14—C18	1.427 (2)	C6—C5	1.401 (2)
O13—C13	1.3769 (18)	C6—H3	0.93
O13—C17	1.425 (2)	C5—C4	1.370 (2)
O5—C5	1.381 (2)	C3—C2	1.357 (2)
O5—C19	1.405 (2)	C3—C9	1.426 (2)
N—C2	1.381 (2)	C3—H9	0.93
N—C8	1.390 (2)	C18—H18A	0.96
N—C10	1.4255 (19)	C18—H18B	0.96
O12—C12	1.3622 (18)	C18—H18C	0.96
O12—C16	1.427 (2)	C4—C9	1.412 (2)
C11—C12	1.384 (2)	C4—H7	0.93
C11—C10	1.387 (2)	C2—H8	0.93
C11—H15	0.93	C16—H16A	0.96
C15—C10	1.382 (2)	C16—H16B	0.96
C15—C14	1.389 (2)	C16—H16C	0.96
C15—H11	0.93	C17—H17A	0.96
C14—C13	1.394 (2)	C17—H17B	0.96
C8—C7	1.393 (2)	C17—H17C	0.96
C8—C9	1.408 (2)	C19—H1A	0.96
C7—C6	1.371 (2)	C19—H1B	0.96
C7—H4	0.93	C19—H1C	0.96
			
C14—O14—C18	117.06 (12)	C2—C3—C9	107.72 (15)
C13—O13—C17	114.63 (14)	C2—C3—H9	126.1
C5—O5—C19	117.49 (16)	C9—C3—H9	126.1
C2—N—C8	108.29 (13)	O14—C18—H18A	109.5
C2—N—C10	125.44 (14)	O14—C18—H18B	109.5
C8—N—C10	126.06 (14)	H18A—C18—H18B	109.5
C12—O12—C16	117.38 (13)	O14—C18—H18C	109.5
C12—C11—C10	119.38 (15)	H18A—C18—H18C	109.5
C12—C11—H15	120.3	H18B—C18—H18C	109.5
C10—C11—H15	120.3	C5—C4—C9	118.11 (15)
C10—C15—C14	119.05 (15)	C5—C4—H7	120.9
C10—C15—H11	120.5	C9—C4—H7	120.9
C14—C15—H11	120.5	C3—C2—N	109.65 (16)
O14—C14—C15	123.64 (14)	C3—C2—H8	125.2
O14—C14—C13	115.72 (14)	N—C2—H8	125.2
C15—C14—C13	120.63 (15)	C8—C9—C4	119.51 (16)
N—C8—C7	130.78 (15)	C8—C9—C3	106.80 (15)
N—C8—C9	107.54 (14)	C4—C9—C3	133.69 (16)
C7—C8—C9	121.66 (15)	O12—C16—H16A	109.5
C6—C7—C8	117.58 (16)	O12—C16—H16B	109.5
C6—C7—H4	121.2	H16A—C16—H16B	109.5
C8—C7—H4	121.2	O12—C16—H16C	109.5
O13—C13—C14	119.30 (15)	H16A—C16—H16C	109.5
O13—C13—C12	121.42 (14)	H16B—C16—H16C	109.5
C14—C13—C12	119.23 (14)	O13—C17—H17A	109.5
O12—C12—C11	123.83 (15)	O13—C17—H17B	109.5
O12—C12—C13	115.82 (14)	H17A—C17—H17B	109.5
C11—C12—C13	120.35 (15)	O13—C17—H17C	109.5
C15—C10—C11	121.32 (15)	H17A—C17—H17C	109.5
C15—C10—N	119.60 (14)	H17B—C17—H17C	109.5
C11—C10—N	119.08 (15)	O5—C19—H1A	109.5
C7—C6—C5	121.68 (17)	O5—C19—H1B	109.5
C7—C6—H3	119.2	H1A—C19—H1B	109.5
C5—C6—H3	119.2	O5—C19—H1C	109.5
C4—C5—O5	125.46 (16)	H1A—C19—H1C	109.5
C4—C5—C6	121.44 (16)	H1B—C19—H1C	109.5
O5—C5—C6	113.10 (16)		

### Table 1 Arene C—H··· arene π interactions between screw-related molecules

Interaction between C4—H of one molecule and the centroid of the six membered (C4 through C9) aromatic ring of the screw-related molecule

**Table:**

H··· ring-centroid distance	Angle between the H···ring-centroid line and the aromatic ring normal
3.035 (1) Å	5.6 (3) °
